# The cost of reducing starting RNA quantity for Illumina BeadArrays: A bead-level dilution experiment

**DOI:** 10.1186/1471-2164-11-540

**Published:** 2010-10-06

**Authors:** Andy G Lynch, James Hadfield, Mark J Dunning, Michelle Osborne, Natalie P Thorne, Simon Tavaré

**Affiliations:** 1 Department of Oncology, University of Cambridge, Li Ka Shing Centre, Robinson Way, Cambridge, CB2 0RE, UK; 2 Cancer Research UK - CRI, Li Ka Shing Centre, Robinson Way, Cambridge, CB2 0RE, UK; 3 Bioinformatics Division, Walter and Eliza Hall Institute of Medical Research, Parkville Victoria 3052, Australia

## Abstract

**Background:**

The demands of microarray expression technologies for quantities of RNA place a limit on the questions they can address. As a consequence, the RNA requirements have reduced over time as technologies have improved. In this paper we investigate the costs of reducing the starting quantity of RNA for the Illumina BeadArray platform. This we do via a dilution data set generated from two reference RNA sources that have become the standard for investigations into microarray and sequencing technologies.

**Results:**

We find that the starting quantity of RNA has an effect on observed intensities despite the fact that the quantity of cRNA being hybridized remains constant. We see a loss of sensitivity when using lower quantities of RNA, but no great rise in the false positive rate. Even with 10 ng of starting RNA, the positive results are reliable although many differentially expressed genes are missed. We see that there is some scope for combining data from samples that have contributed differing quantities of RNA, but note also that sample sizes should increase to compensate for the loss of signal-to-noise when using low quantities of starting RNA.

**Conclusions:**

The BeadArray platform maintains a low false discovery rate even when small amounts of starting RNA are used. In contrast, the sensitivity of the platform drops off noticeably over the same range. Thus, those conducting experiments should not opt for low quantities of starting RNA without consideration of the costs of doing so. The implications for experimental design, and the integration of data from different starting quantities, are complex.

## Background

Gene expression microarrays have become a routine analysis tool; from their introduction [1] to recent headline publications [2-4] their widening use has been primarily down to better understanding of how to design [5,6], use and analyse [7,8] microarray experiments. An important, if somewhat forgotten, design issue has been the amount of starting material needed to produce high quality microarray data. Ten years ago, around 10 *μ*g of total RNA was required and even three years ago many labelling protocols required 1 *μ*g. The introduction of Illumina BeadChips with a standard labelling reaction requiring only 250 ng of total RNA made analysis of some previously unconsidered sample types possible; e.g. limited clinical samples or samples requiring considerable microdissection and pooling.

Whilst many researchers continue to push the limits of starting materials [9], development of robust standard labelling protocols has further decreased the amount of RNA required for microarrays. Until recently 250 ng of starting mRNA was recommended for the Illumina BeadArray platform. Now 50 ng to 100 ng is suggested http://www.illumina.com/technology/direct_hybridization_assay.ilmn. If one can indeed use so little starting material then this is of tremendous importance in terms of the scope of experiments that become possible. However, there is a wealth of literature that is based upon 250 ng, and it is important that future results are comparable to those obtained previously. One small comparison has previously been made [10]. This study found that reproducible signal was obtainable from as little as 25 ng, but the study was not large enough to quantify the costs of such an approach.

Microarray dilution experiments [11,12], where two samples are mixed together in a number of differing (known) ratios and those mixtures hybridized to arrays, have proven to be valuable tools for the comparison and investigation of microarray platforms, most notably in the MAQC project [13]. We employ a nine-level dilution design to investigate the effect of changing the quantity of starting mRNA on the performance of Illumina BeadArrays. We consider the previously recommended level of 250 ng, the current recommended levels of 100 ng and 50 ng and one other (10 ng).

Here, we examine the costs and consequences of reducing the amount of starting RNA, with consideration for the issues of experimental design and meta-analysis, while also providing a unique bead-level dilution experiment to serve as a public resource to the Illumina-using community. We use the Illumina HumanWG-6 V3 BeadArray, analysed at the bead-level as we have previously recommended [14]. One of the benefits of using the bead-level data is that we can analyse separately the two array-sections assigned to any one sample, thus allowing inferences to be made about the more flexible HT12 BeadArrays also. In addition to our purposes, we are creating a unique public resource, and have designed our experiment to be generally useful to the community.

## Methods

### Experimental Design

Six samples can be hybridized to the Illumina HumanWG-6 V3 chip, each sample on two array-sections of approximately 1, 000, 000 beads that are distributed amongst approximately 50, 000 bead-types. We treat the two sections as separate arrays for the purposes of analysis, due to previously observed inter-section differences [14,15]. This also has the effect of making our results comparable to those one might expect from the Illumina HT-12 array which takes 12 samples, allocated one section each.

We have used two reference RNA samples, previously employed in the MAQC study [13], which have subsequently become a standard for microarray [16] and next-generation sequencing [17] studies. These are the Stratagene Universal Human Reference RNA (hereafter "UHRR"), and the Ambion Human Brain Reference RNA (hereafter "Brain"). Nine levels of mixture, including the four employed in the first MAQC study, were then created. These are 100:0, 99:1, 95:5, 90:10, 75:25, 50:50, 25:75, 10:90 and 0:100, where mixtures are presented as UHRR:Brain.

These nine levels allow for investigation of broad trends, and for the detection of subtle differences. Combined with the four levels of starting material that we are investigating, this leads to 36 samples to be arranged across six Illumina HumanWG6 V3 BeadChips. Clearly it would not be desirable to confound levels of starting material with BeadChips as we would be unable to distinguish our comparison of interest from technical variation. However it is desirable that our data resemble data from a 'real-world' experiment else they have no external validity and, in general, experiments are conducted on BeadChips using only one level of starting material.

Our design was chosen to address this tension between internal and external validity. Each BeadChip was run with samples from two starting quantities of RNA (three samples from both chosen starting quantities), and each possible combination of the two starting quantities was run once and only once amongst the six BeadChips. Full details of the design are given in Section 1 of Additional File 1.

### Laboratory methods

Stock UHRR tubes were prepared following manufacturer's recommendation and pooled to create a stock of 1 mg/ml; Brain RNA was received at 1 mg/ml. The quality was checked using the Agilent Bioanalyser. The RNA was accurately diluted to a working stock of 100 ng/*μ*l and the dilution series was created to the specifications given above. The minimum pipetting volume used was 10 *μ*l.

The Illumina TotalPrep-96 Kit (4397949) was used to process the samples using the range of input concentrations in question. For the 50 ng and 10 ng input quantities a 1:10 dilution of working RNA was used. Quality and quantity of the cRNA was checked before proceeding with hybridisation to Human WG-6 V3 BeadArray. The Illumina WGGX DirectHyb Assay Guide (11286331 RevA) protocol was followed for hybridisation, washing and scanning of the BeadArray, with the scanner set to return bead-level data (Additional File 1, Section 2). Quality assessment was achieved via examination of metrics files (Additional File 1, Section 3), agreement with previous MAQC data sets (Additional File 1, Section 4), and performance of housekeeping controls (Additional File 1, Section 5).

### Preprocessing and statistical analysis

Illumina BeadScan files were processed and analysed using the *beadarray *package [18] from Bioconductor. Arrays were pre-processed on the log_2_-scale on a per-array-section basis. BASH [19] was used to remove high-frequency spatial artefacts, followed by outlier removal (outliers being defined as observations more than three median absolute deviations from the median), and expression detection score calculation. The detection score is a standard measure for Illumina expression experiments, and can be viewed as an empirical estimate of the p-value for the null hypothesis that there is no expression. Between-array-section quantile-normalization was performed within each starting material level, and a non-linear regression model fitted across dilution levels within each starting RNA level.

Our approach demands reporting of raw, bead-level, Illumina data, which exceeds the MIAME requirements. As popular repositories are not designed for the storage of raw (bead-level) data from random arrays, the files are available to download from our website at http://www.compbio.group.cam.ac.uk/Resources/Dilution/Dilution.html.

### Statistical model

A previously proposed [20] non-linear model was used as the theoretical model for the dilution curve:

(1) $$E_{mrp}=\log_{2}(c_{m}U_{rp}+(1-c_{m})B_{rp})+\epsilon_{mrp}$$

where *E_mrp _*is the observed (normalized) log-intensity for probe *p *at starting RNA quantity *r *in mixture level *m*, *c_m _*is the proportion of the mixture that is UHRR, *U_rp _*is the intensity associated with probe *p *at starting RNA level *r *in the UHRR sample, and *B_rp _*is similarly defined for the Brain sample. The *ϵ_mrp _*are independent measurement errors with mean zero and standard deviation *σ_rp_*.

This model implicitly assumes a linear relationship between quantity of RNA and measured intensity. This assumption is known not to hold over the full range of observed intensities for microarrays [21], and specifically for Illumina BeadArrays [14]. While some models allow for non-linearity [22], they do not relate it to the known physico-chemical causes. To do so would be difficult and, in any case, would not obviously be advantageous in our situation.

The model can be rewritten in terms of Δ*_rp _*= *U_rp _*- *B_rp_*,

(2) $$E_{mrp}=\log_{2}(c_{m}\;\Delta_{rp}+B_{rp})+\epsilon_{mrp}$$

and we fit this model in R using the nls() function, weighting each observation by the number of beads that contributed to the observation. Under this formulation, it is clear that the test of Δ = 0 from the summary.nls() function in R provides an approximate and quick test of a difference in log-intensities.

### Restricting the analysis-group of bead-types

We have re-annotated the bead-types on the array [23], and have identified 23, 562 "perfect" bead-types (using the August 2009 annotation). These are bead-types that have a full 50 mer match to a reliable transcript, and do not possess additional undesirable properties (e.g. mapping to transcripts masked by repeat regions, having a non-unique transcriptomic match, mapping to transcripts that do not align well to the reference genome, etc.). Additionally, we define an 'analysis-group' of bead-types as a subset of these perfect bead-types that possess two further properties: 1) That their GC content is conducive to hybridization (i.e. in the range of 20-35 bases), which excludes a further 506 bead-types, and 2) That they occur at least six times on each array-section (see Additional File 1, Section 6). All analyses will be restricted to this 'analysis-group' unless otherwise stated.

## Results

### Numbers of beads

The random assembly of Illumina arrays is often a virtue, but prevents the conduct of true replicate experiments. In particular, the number of usable beads on each array can vary, and will influence performance. There are a number of reasons why disparities emerge. Not all beads are decoded by Illumina when the array is manufactured, (which alone leads to the 10 ng experiment having approximately 80, 000 beads more per array-section than the 100 ng experiment). Further beads are 'lost' due to spatial artefacts and to beads being classed as outliers during summarization. The numbers in our experiment are given in Table 1.

**Table 1:** Numbers of beads

Quantity of starting RNA:	250 ng	100 ng	50 ng	10 ng
Total decoded	18,801,235	17,835,076	17,926,750	19,274,434
Removed by BASH	200,459	408,721	284,088	50,449
Removed in summarization	651,323	614,495	603,619	582,345
Remaining	17,949,453	16,811,860	17,039,043	18,641,640
In analysis-group bead-types	7,963,638	7,475,940	7,563,440	8,248,259

Summing across all array-sections in the four experiments, we list the total numbers of beads (as decoded by Illumina), the numbers we remove as being in spatial artefacts using BASH [19], those removed as being outliers in the summarization, the remainders, and the numbers remaining that lie in the analysis-group of bead-types.

It has been observed previously that spatial artefacts can be associated with nearby regions where beads are non-decoded [24], so it may not be coincidental that the experiment with the greatest number of beads loses fewest to spatial artefacts. The differing numbers of beads may cause concern, although it should be noted that the median number of replicates for a bead-type only varies from 21 for the 10 ng experiment to a still very healthy 19 for the 100 ng experiment. The lack of monotonicity is also helpful; the trends that we show do not correlate with the total bead-numbers, suggesting that these numbers are not driving the results. Whilst we take 250 ng of starting RNA as our gold standard for comparison, we can also gain reassurance through comparisons to the 100 ng experiment which contained fewest beads.

As noted above, we restrict analyses to an analysis-group containing only 'perfect' bead-types, with desirable GC composition and at least six beads on each array-section. This reduces the number of bead-types considered to 21, 627. This also has a marginal effect on improving the balance between experiments in terms of the numbers of beads analysed.

### Detection of expression

In Table 2 is presented a summary of agreement between experiments for the detection of expression (using a significance level of < 0.01) for the analysis-group (see also Additional File 1, Section 7). If no bead-types were truly expressed, we would expect to see 3, 579 apparently showing expression in at least one array-section and nine showing expression in both UHRR and brain. Even acknowledging this, we see that a substantial number of the analysis-group show expression above negative-control levels.

**Table 2:** Expression detected

Quantity of starting RNA:	250 ng	100 ng	50 ng	10 ng
...at least one array-section	15,880	15,597	15,691	14,090
...both UHRR and Brain	11,992	11,248	10,965	8,775
...all array-sections	9,964	9,178	8,996	5,975
mean number of array-sections	6.94	6.59	6.47	4.95

For each of the four experiments, we report the number of analysis-group bead-types for which expression was detected in at least one of the 18 array-sections, at least one of the two 100% UHRR array-sections and at least one of the two 100% Brain array-sections, and in all 18 array-sections. Additionally for the analysis-group bead-types, we report the mean number of array-sections out of 18 in which expression is detected.

Naturally, any bead-type that shows expression in both Brain and UHRR should show expression in all mixtures of those two samples, and we see that the proportion of bead-types satisfying the former that are also returned by the latter exceeds 80% for the 250 ng, 100 ng and 50 ng experiment but reduces to below 70% for the 10 ng experiment. Agreement between experiments is reported in Table 3, and is encouraging. Performance in terms of sensitivity while not perfect at 100 ng only decreases dramatically when we reach 10 ng, but here still returns 2/3 of the bead-types that were detected in both UHRR and Brain using 250 ng. Notably, the false discovery rate is fairly constant, staying below 10% even at 10 ng. Thus while one will detect expression in fewer bead-types using less starting RNA, the validity of that which is detected is preserved.

**Table 3:** Consistency in expression detection between quantities of starting RNA

	reference experiment		
**test experiment**	**250 ng**	**100 ng**	**50 ng**
10 ng	0.67/0.09	0.71/0.09	0.73/0.09
50 ng	0.86/0.06	0.89/0.09	
100 ng	0.86/0.08		

For the numbers of bead-types with detected expression in both 100% Brain and 100% UHRR reporting "X/Y" where X is the proportion of bead-types reported for the reference experiment also reported for the test experiment (e.g. a measure of sensitivity), and Y is the proportion of bead-types reported by the test experiment that were not reported by the reference experiment (e.g. FDR). So for example, taking 250 ng as a gold-standard, for this detection measure the 100 ng experiment has 86% sensitivity and an FDR of 0.08.

### Expression of control bead-types

The detection p-values for expression depend on the performance of negative control bead-types for their calculation. This platform has 759 negative control bead-types, which should have no match to the human transcriptome. Due to the nature of the calculation, at least seven (1%) of these will themselves apparently detect significant expression. Table 4 summarizes the numbers seen in our experiments. We see markedly more than seven negative control bead-types being called as 'detected', and far more than would be expected by chance being consistently called as detected.

**Table 4:** Control bead-type summary

Quantity of starting RNA:	250 ng	100 ng	50 ng	10 ng
Negative controls detected in UHRR	37	32	20	0
Negative controls detected in Brain	35	40	29	24
Negative controls detected in all array-sections	10	6	2	0
Negative controls detected in at least one array-section	88	89	98	99
Negative controls: median log-intensity UHRR	5.78	5.79	5.73	5.58
Negative controls: median log-intensity Brain	5.80	5.82	5.72	5.58
Housekeeping controls: median log-intensity UHRR	13.46	13.14	13.09	11.32
Housekeeping controls: median log-intensity Brain	12.47	12.52	12.03	10.64

Giving a) the numbers of the 759 negative-control bead-types that show expression in one or more array-sections, and b) median log-intensities for negative-control and housekeeping bead-types in 100% UHRR and 100% Brain array-sections.

Such observations could have explanation other than the bead-types showing specific signal. For instance, thermodynamic variation could lead to some negative control bead-types regularly being called as 'detected', but evidence of differential expression is harder to explain. Using Benjamini-Hochberg control for false discovery rate, there are still three negative control bead-types for the 250 ng starting material experiment (two for 100 ng, eight for 50 ng, and four for 10 ng) that show differential expression. The greatest evidence of a negative control showing specific hybridization is for bead-type ILMN_1343923 (Additional File 1, Section 8).

**Figure 1:**
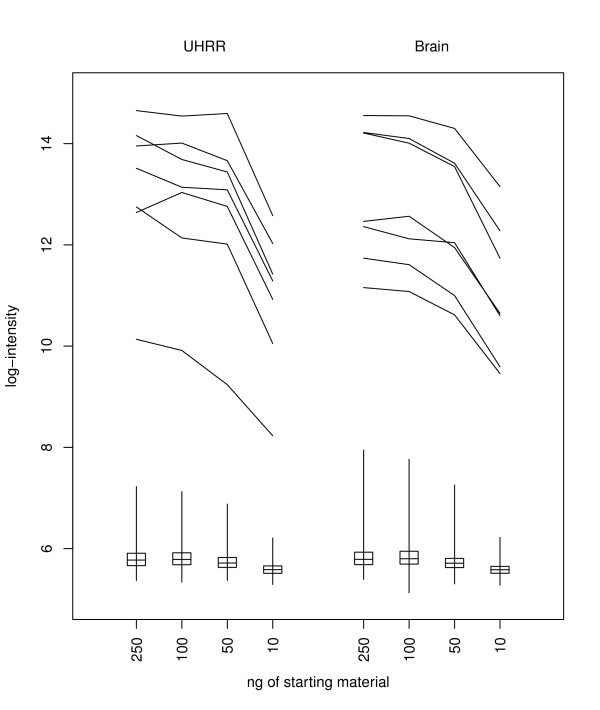
**The change in control bead-type performance with quantity of starting RNA**. Illustrating for array-sections hybridized to either 100% Brain or 100% UHRR, the log_2_-intensities seen for selected control bead-types. The medians, ranges and interquartile ranges of the 759 negative control bead-types are illustrated with box and whisker plots, while the profiles of the seven housekeeping controls are also indicated.

The amount of starting RNA varies between experiments, but the amount of cRNA used is the same in every case, so there is no reason to anticipate overall changes in intensity levels. However, the intensity levels change for both the housekeeping bead-types (bead-types that target genes EEF1A1, GAPDH, TXN, ACTB, TUBB2A, RPS9, UBC) and the negative control bead-types, suggesting that the levels of non-specific hybridization vary according to the amount of starting material (Table 4, Figure 1).

The log-intensity levels for the housekeeping control bead-types decrease at a greater rate than those for the negative control bead-types (except when saturation effects are apparent). Thus the log-fold-change in intensities from housekeeping gene to negative control (a measure of signal to noise) decreases with the amount of starting RNA. This change in performance is apparent even at 100 ng levels of starting material. Other control bead-types on the Illumina BeadArray platform are not sample dependent, and do not vary considerably between starting quantities of RNA.

### Magnitude of expression

**Figure 2:**
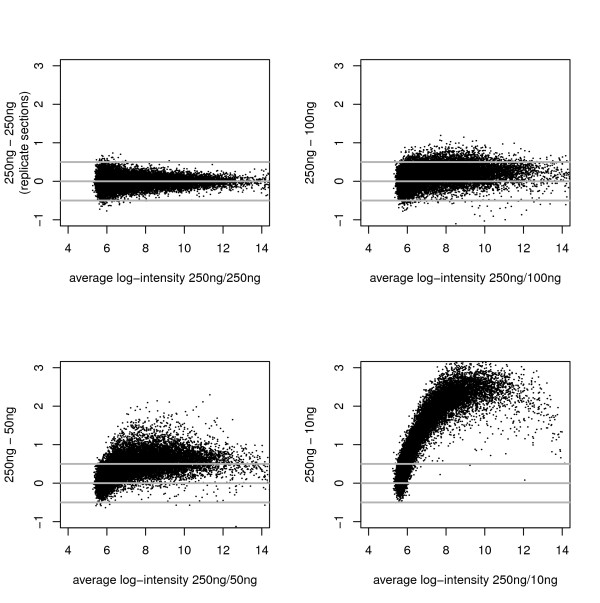
**The change in expression with quantity of starting RNA**. Depicted are "MA" plots where the difference of two log_2_-intensities (y-axis) is plotted against the average of the log_2_-intensities. Here we show the agreement of log_2_-intensity between a section of the 250 ng experiment and all four levels of starting RNA (using replicate array-sections to make the 250 ng vs 250 ng comparison).

Figure 2 shows MA plots for the analysis-group of bead-types comparing single array-sections of 100% UHRR. Although true agreement with intensities from 250 ng is clearly poor when small amounts of starting material are used, the rank-correlation between array-sections remains high even for 10 ng of starting RNA (Table 5), suggesting that it may be possible to normalize samples arising from different starting levels of RNA.

**Table 5:** Squared rank correlations

	Quantity of starting RNA			
	**250 ng**	**100 ng**	**50 ng**	**10 ng**
250 ng	0.954	0.933	0.921	0.784
100 ng		0.933	0.916	0.789
50 ng			0.924	0.784
10 ng				0.797

Giving the square of Spearman's rank correlation for the intensities of the analysis-group bead-types between 100% UHRR array-sections

It is also clear from Figure 2 that intensities generally decrease with the quantity of starting RNA, as was observed specifically for the control bead-types. This loss of signal leads to an apparent diminishing of technical biases (e.g. if all signal were lost then we would cease to observe the diminishment of signal as target locations become more 5' along the gene), which should not be mistaken for a benefit.

### Differential expression

The number of bead-types identified as showing differential expression (*p *< 0.001, for the non-linear model), decreases with the amount of starting material much as did the number for which expression was detected (Table 6). Naturally, differential expression implies expression, so we might expect to see the numbers for differential expression bounded by the numbers we saw for expression. The decline in numbers of bead-types for which differential expression is noted is more marked than would be required simply by this constraint. Moreover we should note that due to the nature of the two tests, it is entirely possible to detect differential expression across the set of array-sections, but not detect expression in any individual array-section (Additional File 1, Section 9): evidence that the filtering of bead-types based on expression-detection scores requires caution.

**Table 6:** Differential expression detected

	250 ng	100 ng	50 ng	10 ng
amongst all bead-types	15,753 (32%)	14,361 (29%)	13,741 (28%)	9,579 (19%)
amongst analysis group	11,021 (51%)	10,169 (47%)	9,788 (45%)	7,084 (33%)

Showing the numbers (and percentage) of bead-types from the complete set of 49,575 for which differential expression between Brain and UHRR was detected. Also showing the same measures for the analysis-group bead-types.

**Figure 3:**
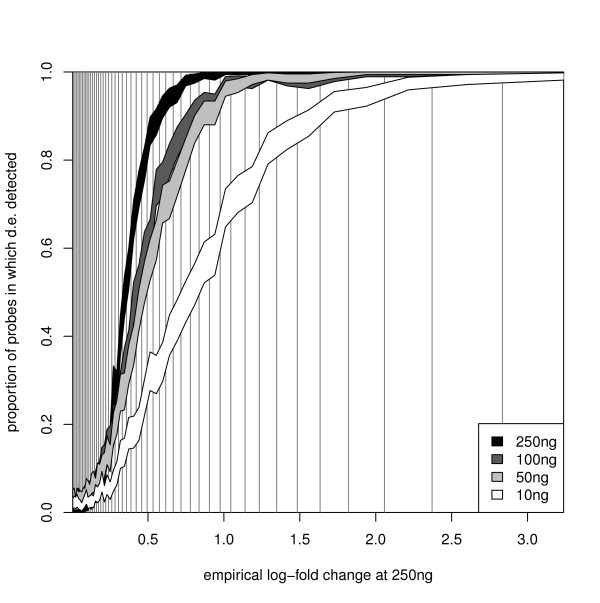
**The power to detect differential expression by quantity of starting RNA**. Illustrating, for the analysis-group of bead-types, the increased ability to detect large log_2_-fold changes (for all levels of starting RNA), and how the relationship (between that ability and the size of the log-fold change) varies with the quantity of starting RNA. The empirical log fold change calculated from the 250 ng experiment is depicted on the x-axis, which is divided into 50 bins, each containing 2% of the bead-types (indicated by the vertical lines). On the y-axis are indicated 95% confidence intervals for the proportion of bead-types in each bin for which differential expression will be detected.

Once more, the sensitivity (defined as for expression detection) is high with a drop-off only when 10 ng of starting RNA are used, and the FDR (defined as for expression detection) remains low across all quantities of starting RNA (Table 7). If we break down the comparison by the magnitude of differential expression (taking 250 ng as the gold standard and comparing the log-expression between 100% UHRR and 100% Brain), then it is apparent (Figure 3) that one pays a price for using the 10 ng level of starting material across the full range of log-fold changes (Additional File 1, Section 10). The performance of the 100 ng and 50 ng starting levels is better, and matches 250 ng outside the range of 0.25 to 1.25. Within that range, they return a lower proportion of bead-types as being differentially expressed, while the 100 ng level of starting material also outperforms the 50 ng level.

**Table 7:** Consistency in detection of differential expression between quantities of starting RNA

	reference experiment		
**test experiment**	**250 ng**	**100 ng**	**50 ng**
10 ng	0.62/0.04	0.67/0.04	0.69/0.05
50 ng	0.83/0.07	0.88/0.08	
100 ng	0.86/0.06		

For the numbers of bead-types for which differential expression between UHRR and Brain was detected we report "X/Y" where X is the proportion of bead-types reported for the reference experiment also reported for the test experiment (e.g. a measure sensitivity), and Y is the proportion of bead-types reported by the test experiment that were not reported by the reference experiment (e.g. FDR). So for example, taking 250 ng as a gold-standard, for this detection measure the 100 ng experiment has 86% sensitivity and an FDR of 0.06.

### Signal to noise

The variance of observations is not independent of their value. Since expression levels decrease as the quantity of starting RNA decreases, it is not possible to assess the change in variance as the quantity of starting RNA decreases, without simultaneously considering the level of expression.

From the non-linear model we can compare the estimate of the difference in expression levels to the estimated standard error of the difference. This side-steps the complications of the variance and fluorescence levels changing in a dependent manner as the amount of starting material changes. Considering only the analysis-group of bead-types, the median ratios of standard error to estimate are 0.23, 0.28, 0.31 and 0.52 for 250 ng, 100 ng, 50 ng and 10 ng of starting RNA respectively. The median ratios of the two signal to noise ratios are 1.12, 1.16 and 1.76 for 100 ng, 50 ng or 10 ng respectively comparing to a reference starting quantity of 250 ng.

## Discussion

### Meta-analysis

Inevitably, there will be occasions when we wish to combine data sets generated using different quantities of starting material, possibly because we are performing a meta-analysis across different experiments, or possibly because not all samples within a single experiment can supply the desired quantity of starting RNA. Our analysis has, so far, considered the different quantities of starting material in this study as being different experiments, but we will now briefly consider strategies for combining them.

Consider if samples were run in strata of starting RNA, e.g. we have an experiment where some samples were run using 250 ng, while others were run using 50 ng. The strata were not balanced in terms of experimental design, so we may not wish to obtain two simple estimates for the parameters of interest (one from each stratum) and then combine the estimates. Our strategy for analysis may depend on whether some samples had been run in both strata.

Consider further that we only have Brain run at 50 ng and UHRR at 250 ng, and we wish to transform the 50 ng Brain data for comparison with UHRR. Essentially we wish to simulate a 250 ng Brain data set from this restricted data set, and can use the fact that we do have Brain run at 250 ng to assess the performance. We will consider both the scenario where we have only the two samples with which to work, and a second where we have additionally run UHRR at 50 ng.

**Figure 4:**
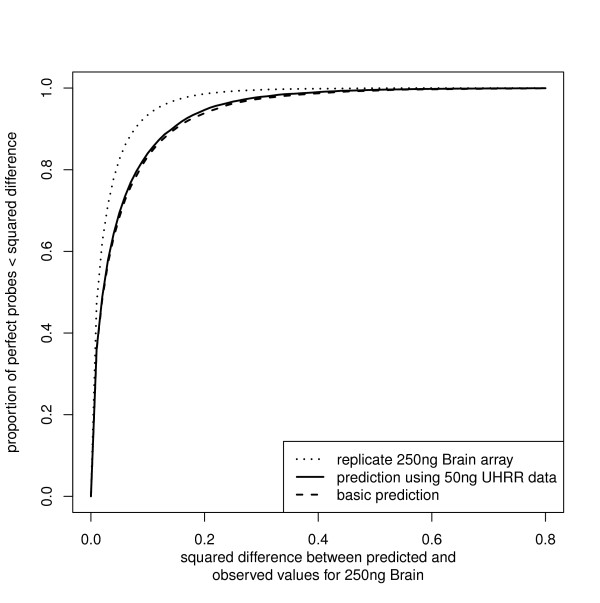
**The performance of alternative normalization strategies**. The performances of different strategies for normalizing a 50 ng Brain array-section for comparison to a 250 ng UHRR array-section are illustrated. For the analysis-group bead-types, with a 250 ng Brain array-section as a reference, we determine the squared differences between our prediction of log_2_-intensity and the reference and illustrate the cumulative distribution of those differences. The first prediction uses a basic quantile-style normalization where the 50 ng Brain array-section is transformed to take the distribution of intensities seen on the 250 ng UHRR array-section. A more complicated prediction making use of a 50 ng UHRR section is also illustrated. For reference we give the agreement between replicate 250 ng Brain array-sections, representing the gold-standard that could be achieved by any method.

If we are in this first scenario, then there is little option but to normalize between the samples. The high rank correlation we have observed between data arising from different starting amounts of RNA gives cause for optimism that a simple quantile-style normalization of the 50 ng data to the expression profile of the 250 ng data will prove successful. With data available from samples run at both starting levels, we can use the 50 ng UHRR and 250 ng UHRR samples to estimate the bias due to starting RNA quantity (via fitting a locally smooth regression) and can then project the 50 ng Brain sample with that model to obtain our prediction for how a 250 ng Brain sample would look. Such an approach shows a marginal improvement over the basic attempt in our example (Figure 4).

Using the additional data (50 ng UHRR) makes only a small improvement to our ability to transform the 50 ng Brain data and in a real experiment running 50 ng of an additional sample may provide greater value to the ultimate analysis. We should be wary of trying to use a sample for both bias-estimation and analysis as there will be a lack of independence between these samples and all those that are bias-corrected using the results. Moreover the small improvement we see here, over the simpler quantile-normalization style approach, comes using samples that have large numbers of expressed genes. For bias correction of this nature to be useful, we need to observe a wide range of log-intensities which in turn requires large numbers of genes to be expressed. Thus the appropriateness of this more complicated method will be dependent on the size of the experiment and the nature of the samples being hybridized.

### Implications for experimental design

A number of implications for experimental design are obvious. It is clear that all things being equal, of the range of starting quantities of RNA considered here, it is preferable to use 250 ng. If there are limitations to the amount of starting RNA available, then the more starting material used the better (within this range examined). Should the amount of available RNA differ between samples then more subtle decisions are required. On the basis of the signal-to-noise results, we can infer that if using 100 ng or 50 ng then the sample size would need inflating by a factor of at least 1.2 to achieve the same performance, while if using 10 ng, then in the region of three times the numbers will be required. Thus, when we have the choice and free from other pressures, reducing the starting RNA level is only desirable if it allows sample numbers to be increased by these factors.

The combination of multiple starting RNA levels in one experiment will be problematic. If we wish to normalize using data from the same sample hybridized from multiple quantities of starting RNA, then clearly we must stratify samples into a few starting quantities. If we do not have, or do not wish to make, recourse to replicate samples hybridized from several RNA quantities, and are simply going to normalize samples together, then there is merit in using as much starting RNA as possible for each sample, as was noted in the previous section.

In this scenario, where all samples are independent, it would still be hard to criticize a design that opted for a fixed number of starting levels, especially if this came at minimal cost to quality (i.e. 250 ng reduced to 220 ng but not to 10 ng) and allowed balance of experimental criteria to be achieved within each stratum of starting quantity. Such an approach is suboptimal by our criterion, but may be more robust to those unexpected events that befall real-world experiments.

## Conclusions

We have presented a bead-level Illumina BeadArray dilution control experiment that will be a valuable resource for the Illumina analysis community. As intended, the experiment also answers an important experimental question regarding the required levels of starting RNA, however it also allows for a number of questions to be addressed regarding experimental design when large quantities of RNA are difficult to obtain.

We have shown that reliable signal is obtainable using as little as 10 ng of starting RNA. However we have also seen that lower levels of starting RNA are associated with a bias in expression levels (which may be correctable), and drop in sensitivity (which will not be).

This increase in noise implies that, if using less starting RNA, more samples would be needed in an experiment to achieve the same levels of precision. However, it seems that few false discoveries result from using even as little as 10 ng of starting RNA. Thus while a small experiment using a low starting quantity of RNA may fail to identify many subtle changes, one can have confidence in any changes that are reported.

## Authors' contributions

AGL finalized the design of the experiment, performed the analysis, and drafted the manuscript. JH supervised the experiment and drafted the manuscript. MJD participated in the design of the study, participated in the array data processing and provided Illumina expertise. MO conducted the experiment. NPT and ST participated in the conception and design of the study. All authors read and approved the final manuscript.

## Supplementary Material

Additional file 1 **Supplementary material**. File giving details of 1) Experimental Design: Array Layout, 2) Lab Methods: Obtaining bead-level data, 3) Lab Methods: Quality assessment metrics, 4) Lab Methods: Quality assessment - comparison with MAQC, 5) Lab Methods: Quality assessment - Association between starting RNA quantity and intensity, 6) Criteria for including bead-types, 7) Results: Detection, 8) Results: Negative controls, 9) Results: Differential expression but no expression, and 10) Results: Differential expression - detection of small changes. Click here for file
