# Supplementary material for: The cost of reducing starting RNA quantity for Illumina BeadArrays: A bead-level dilution experiment
Source: BMC Genomics. 2010 Oct 6;11:540. doi: 10.1186/1471-2164-11-540 (PMC3091689; doi:10.1186/1471-2164-11-540)
Supplement: Additional file 1 — Supplementary material. File giving details of 1) Experimental Design: Array Layout, 2) Lab Methods: Obtaining bead-level data, 3) Lab Methods: Quality assessment metrics, 4) Lab Methods: Quality assessment - comparison with MAQC, 5) Lab Methods: Quality assessment - Association between starting RNA quantity and intensity, 6) Criteria for including bead-types, 7) Results: Detection, 8) Results: Negative controls, 9) Results: Differential expression but no expression, and 10) Results: Differential expression - detection of small changes. [file 1471-2164-11-540-S1.PDF]

Supplementary information for “The cost of reducing starting RNA quantity for Illumina BeadArrays: A bead-level dilution experiment.”

Andy G Lynch, James Hadfield, Mark J Dunning, Michelle Osborne, Natalie P Thorne and Simon Tavaré

Abstract

- 1 - Experimental Design: Array Layout (Page 2)
- 2 - Lab Methods: Obtaining bead-level data (Page 4)
- 3 - Lab Methods: Quality assessment metrics (Page 5)
- 4 - Lab Methods: Quality assessment - comparison with MAQC (Page 6)
- 5 - Lab Methods: Quality assessment - Association between starting RNA quantity and intensity (Page 9)
- 6 - Criteria for including bead-types (Page 10)
- 7 - Results: Detection (Page 11)
- 8 - Results: Negative controls (Page 12)
- 9 - Results: Differential expression but no expression (Page 14)
- 10 - Results: Differential expression - detection of small changes (Page 15)

---

1 Experimental Design: Array layout

Nine different mixtures of RNA were used, with 4 different starting quantities employed, giving 36 arrays (necessitating 6 Illumina HumanWG6 V3 BeadChips).

Concerns about between-chip variability lead to a tension between wishing not to confound the starting quantity with BeadChips (so as to facilitate a fair comparison of starting quantities – maximizing the internal validity of the experiment) while maximizing the utility of the data (where we might anticipate users only wanting to look at one starting quantity in which case we would not want any dilution levels confounded with BeadChips – maximizing the external validity).

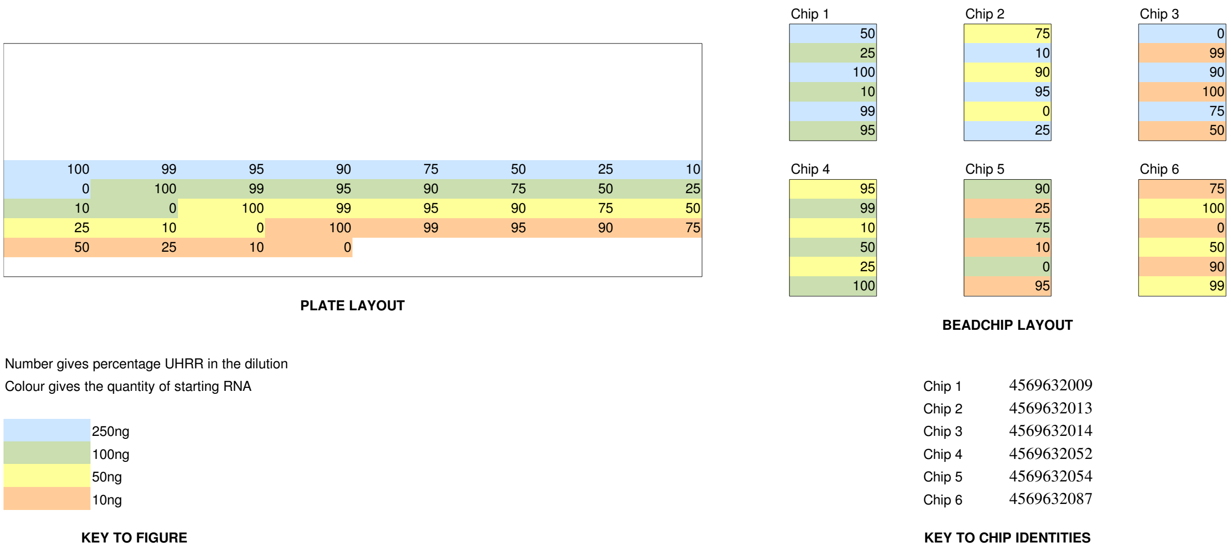

Figure A1: Showing the manner in which samples were laid out on the BeadChips.

The design we have chosen is a compromise for this tension between internal and external validities, and has the following features:

- Each starting quantity of RNA appears on a bead-chip with each other starting quantity of RNA exactly once.
- When a starting quantity appears on a bead-chip, it appears in three arrays on the chip – allowing the dilution curve to be estimated within a chip if so desired, and a single starting quantity to be studied. Thus the nine dilution levels are divided into three sets of three.
- The three dilution levels that appear on the chip for one starting quantity are chosen such that they contain one pair of levels that are near to each other (to allow investigation of sensitivity) and one level that is further away (to allow better estimation of the dilution curve within a chip).

- The three dilution levels on the array for one starting quantity of RNA will not be the same as the three starting levels on the array for the second starting quantity that features, but the range of the two dilution curves that can be estimated will overlap.

- The dilution levels on a chip are not monotonically ordered.

2 Lab Methods: Obtaining bead-level data

Bead-level data were obtained by adjusting the settings.xml file in the Illumina BeadScan directory in a manner documented at http://www.compbio.group.cam.ac.uk/Resources/illumina/index.html.

Essentially the following tags were set to true:

- <GenerateVersionTwoIdatFiles>
- <SavePerBeadFiles>
- <SaveTextFiles>
- <IncludeXY>

The following tag was set to false:

- <ExcludeOutliers>

3 Lab Methods: Quality assessment metrics

Illumina output some standard metrics to a text file upon the scanning of a BeadChip. We do not make use of these in this document, but they are given in Table A1 for completeness. Of particular note may be the 5th and 95th intensity percentiles respectively (P05 and P95). These cannot be interpreted absolutely, as they are highly dependent on scanner settings, but within this experiment, relative patterns can be informative. For further information regarding Illumina's quality control tools, see 'Technical Note: "Gene Expression Microarray Data Quality Control"'.

Table A1: Illumina's quality metrics as returned by the scanner

Matrix	Section	Reg	Focus	P95	P05	Matrix	Section	Reg	Focus	P95	P05
4569632009	A_1	1	0.59	1214	49	4569632052	A_1	0.1	0.58	831	48
4569632009	A_2	0.11	0.43	1256	50	4569632052	A_2	0.11	0.42	716	45
4569632009	B_1	0.52	0.7	1085	51	4569632052	B_1	0.84	0.67	1045	48
4569632009	B_2	1	0.68	1075	50	4569632052	B_2	1	0.66	1068	49
4569632009	C_1	1	0.69	1312	49	4569632052	C_1	1	0.45	588	44
4569632009	C_2	0.11	0.7	1375	50	4569632052	C_2	0.16	0.16	676	49
4569632009	D_1	0.13	0.68	722	45	4569632052	D_1	0.11	0.34	1080	52
4569632009	D_2	0.13	0.65	758	45	4569632052	D_2	1	0.55	984	49
4569632009	E_1	0.47	0.69	1171	51	4569632052	E_1	0.15	0.61	546	47
4569632009	E_2	1	0.68	1175	51	4569632052	E_2	0.1	0.57	551	46
4569632009	F_1	0.44	0.67	1088	50	4569632052	F_1	1	0.64	1069	51
4569632009	F_2	1	0.65	1099	50	4569632052	F_2	1	0.61	1114	53
4569632013	A_1	0.12	0.66	794	48	4569632054	A_1	0.1	0.67	889	45
4569632013	A_2	0.12	0.68	848	48	4569632054	A_2	0.12	0.6	999	50
4569632013	B_1	0.12	0.65	1013	47	4569632054	B_1	0.2	0.31	181	43
4569632013	B_2	0.13	0.66	1036	49	4569632054	B_2	0.09	0.43	186	45
4569632013	C_1	0.12	0.65	803	46	4569632054	C_1	0.11	0.68	900	47
4569632013	C_2	0.12	0.59	753	46	4569632054	C_2	0.11	0.65	975	50
4569632013	D_1	0.1	0.67	1181	48	4569632054	D_1	0.07	0.13	193	44
4569632013	D_2	0.1	0.67	1260	48	4569632054	D_2	0.23	0.57	203	45
4569632013	E_1	0.1	0.64	613	46	4569632054	E_1	1	0.65	841	49
4569632013	E_2	0.15	0.64	581	45	4569632054	E_2	1	0.64	886	51
4569632013	F_1	0.14	0.66	1019	48	4569632054	F_1	1	0.6	226	45
4569632013	F_2	0.11	0.65	1139	50	4569632054	F_2	0.17	0.27	239	44
4569632014	A_1	0.39	0.7	1039	47	4569632087	A_1	0.16	0.66	262	46
4569632014	A_2	0.12	0.7	1018	48	4569632087	A_2	0.16	0.57	273	46
4569632014	B_1	0.17	0.63	256	43	4569632087	B_1	1	0.54	858	52
4569632014	B_2	0.16	0.61	240	42	4569632087	B_2	1	0.67	904	52
4569632014	C_1	1	0.69	1218	49	4569632087	C_1	1	0.53	218	44
4569632014	C_2	0.1	0.69	1142	48	4569632087	C_2	0.15	0.61	237	46
4569632014	D_1	1	0.63	249	44	4569632087	D_1	1	0.6	857	50
4569632014	D_2	0.14	0.61	255	45	4569632087	D_2	0.14	0.65	835	47
4569632014	E_1	1	0.68	1180	51	4569632087	E_1	0.16	0.62	222	45
4569632014	E_2	0.11	0.66	1080	49	4569632087	E_2	0.18	0.45	217	44
4569632014	F_1	0.17	0.62	208	44	4569632087	F_1	0.11	0.52	836	51
4569632014	F_2	0.08	0.58	222	45	4569632087	F_2	0.12	0.5	883	51

4 Lab Methods: Quality assessment - comparison with MAQC

We compare our intensities with those from the original MAQC study [see reference 13 in main article], using the normalized datafile norm_MAQC_ILM_123_qNorm16.zip as detailed in the document "Summary of the MAQC Data Sets" (obtainable from http://edkb.fda.gov/MAQC/MainStudy/upload/Summary_MAQC_DataSets.pdf). While there are differences in the technology used (MAQC used version 1 Illumina arrays, we used version 3) and the processing methods employed, we see reasonable concordance with the data produced from our gold-standard (250ng of starting material) dilution experiment.

Using our Aug09 annotations (available from http://www.compbio.group.cam.ac.uk/Resources/Annotation/index.html) for the two platforms, we find 6113 bead-type sequences on both platforms that we rate as "perfect" and which have GC content in the 40% to 70% band. Of course, we should note that we cannot be sure that the decoding sequences used in these bead-types do not differ between platforms, and also that the differing contents of the platforms will affect the values of the common contents through the normalization steps. Nevertheless, we take the values of these 6113 bead-types as being comparable between platforms.

The original MAQC study considered 4 dilution levels of which ours are a superset. Characterizing these 4 in terms of their percentage UHRR we can denote them 100%, 75%, 25% and 0%. We construct two sets of log-ratios: 100%-25% and 75%-0% that will demonstrate only subtle differences even in bead-types that show evidence of expression. Essentially, if U and B are the intensities associated with 100% UHRR and 100% Brain, and linearity between DNA quantity and intensity holds, then we are comparing the two log-ratios $\log_2(\frac{100U}{25U+75B})$ and $\log_2(\frac{75U+25B}{100B})$.

In Figure A2 we see that, for one set of dilutions from the MAQC study and for one set from our study, there is broad agreement for the majority of bead-types (5386 in the indicated region of magnitude < 1.5). It is only the non-linearity of the relationship between the two log-ratios in the tails of the relationship that will allow us to distinguish between the two sets of log-ratios.

Immediately, we can note that the agreement between these two laboratories in terms of this non-linearity (and, by extension, the pattern of response of cDNA quantity to intensity in the two laboratories is good).

More generally, we can cluster (using complete clustering on Euclidean distance) the 33 sets of 6113 log-ratios. 17 sets are 100% vs 25% (5 from each of the three MAQC centres + our two from the CRI) and 16 are 75% vs 0% (MAQC centre 1 only provides 4 replicates for this comparison). This is illustrated in Figure A3, where we see that the log-ratios calculated from 100% - 25% UHRR levels separate completely

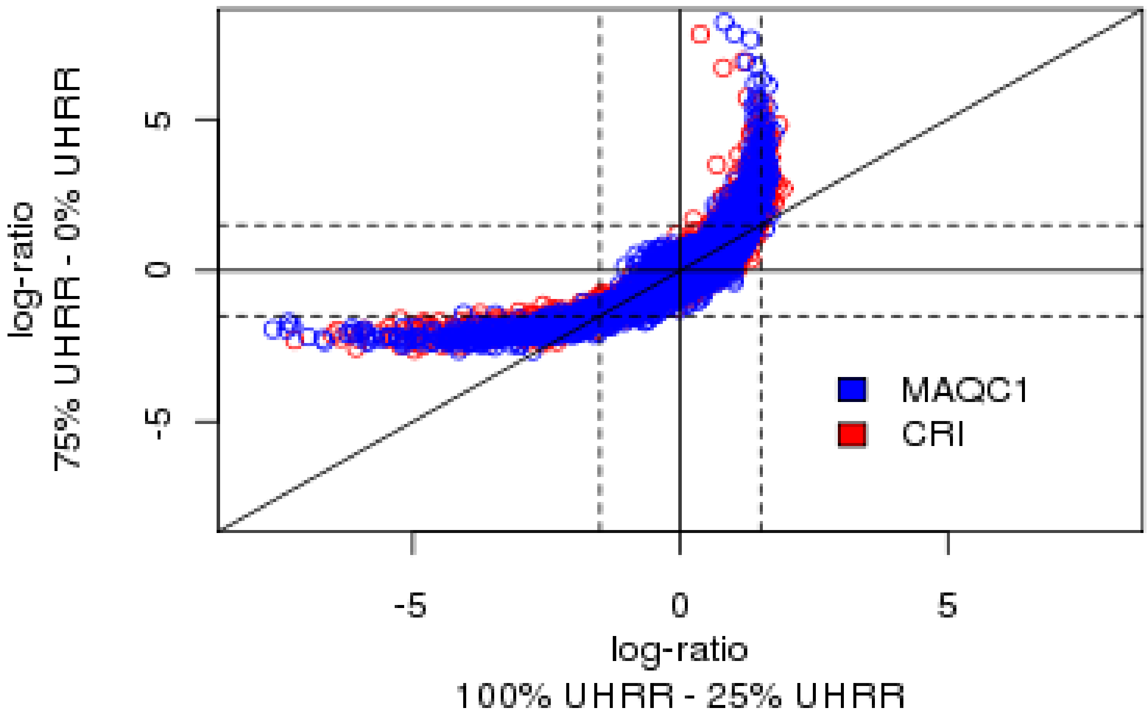

Figure A2: Scatterplot comparing the two log-ratios under consideration. One log-ratio is generated from the 100% UHRR and 25% UHRR dilution levels, while the other is generated from the 75% UHRR and 0% UHRR dilution levels. The relationship is shown for one of the MAQC replicates and one of our (CRI) replicates.

from the 75% - 0% UHRR levels. Mixing between centres is more complete amongst the 100% - 25% UHRR log-ratios, but even in the 75% - 0% UHRR log-ratios where our two replicates (CRI) cluster together, they do so within the replicates from MAQC centre 3.

It would be a serious concern if the technical variation between sites could overcome the minimal biological variation between the log-ratios and happily it does not. Nor do we find that within one set of log-ratios that the CRI values cluster apart from the MAQC values. In view of the technical differences between the studies, we find this consistency a reassurance that the data we have produced are broadly comparable to those from the MAQC study.

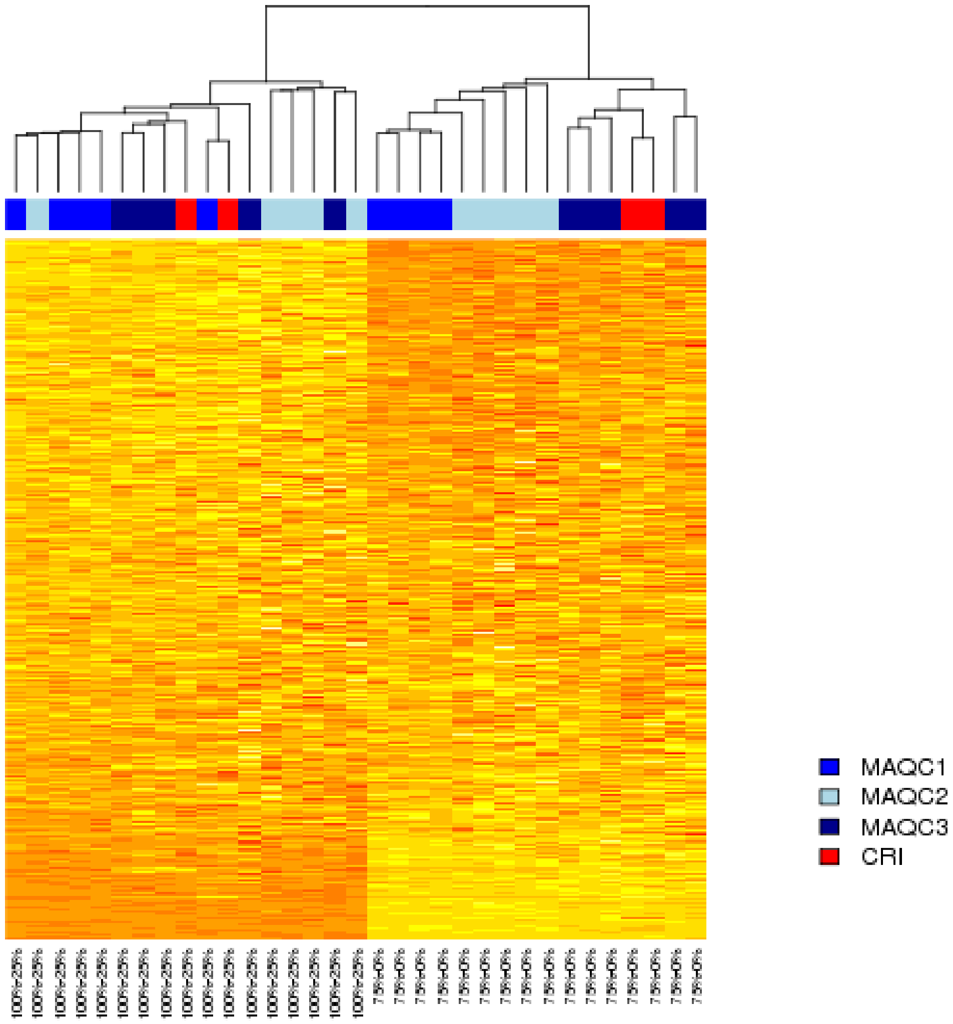

Figure A3: Clustering and heatmap for 33 sets of log-ratios across 6113 bead-types. The clustering is performed as described in the above text, while each row of the heatmap is centred and scaled so while yellows indicate high relative values for a bead-type and reds indicate low relative values, the colours have no absolute meaning.

5 Lab Methods: Quality assessment - Association between starting RNA quantity and intensity

Plotting the logarithms (to base 2) of raw intensities associated with a housekeeping control bead-type ILMN_2038777 (targeting the 3′ UTR of ACTB) from all arrays we see that expression is monotonically associated with starting RNA quantity. While there are some between-chip effects, within a chip the values associated with the greater quantity of starting material always show greater intensity. Note that the numbers of observations of this bead-type are relatively high with a median of 36 beads per array-section for the 250ng, 100ng and 50ng experiments and 44.5 beads per array-section for the 10ng experiment. Across the entire experiment the number of beads per array section, for this bead-type, varied from 26 to 52.

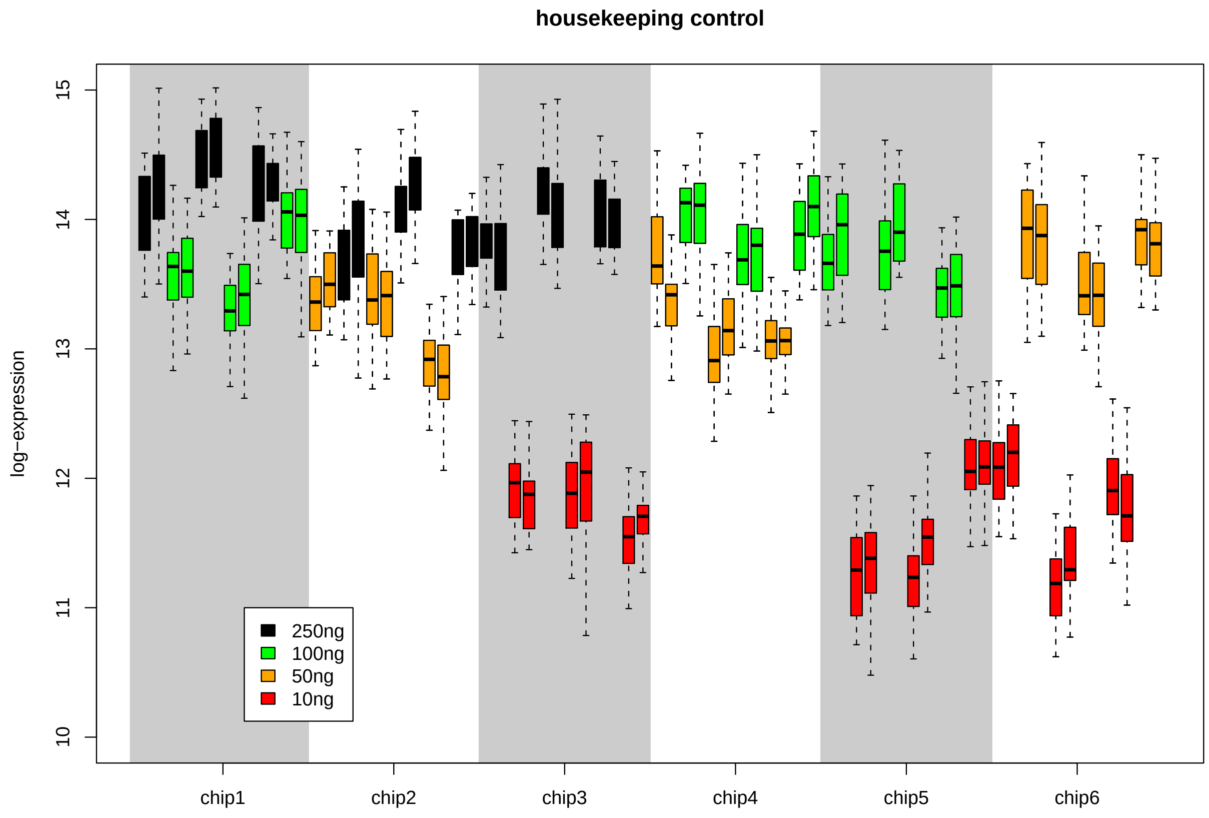

Figure A4: Illustrating the ACTB housekeeping control bead-type intensities for each array section in the experiment.

6 Methods: Criteria for restricting the bead-types used for analysis

We use three criteria for including a bead-type in the analysis-group for this paper: (1) The quality score from the annotation [reference 16 in the main manuscript], (2) the GC contents of the bead-type, and (3) the minimum number of beads across the 72 array-sections in the experiment. Each of these is illustrated in Figure A5.

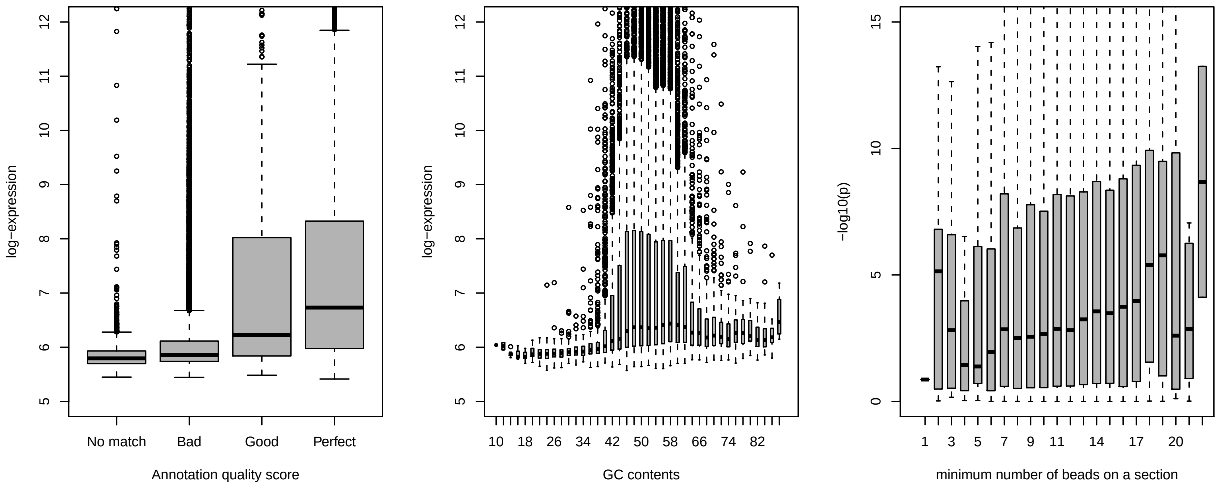

Figure A5: Illustrating the criteria for inclusion of bead-types.

The annotation quality score is clearly associated with $\log_2$-expression. “Bad” bead-types can be highly expressed (often because they cover repeat-masked elements or have multiple targets), but are generally poor bead-types. “Good” bead-types have a performance much closer to that of the “perfect” bead-types, and could have been included, but they are still markedly worse in performance and we choose not to do so.

From other data sets [not shown] we have noted that bead-types outside the range of 40% to 70% GC contents do not show expression. A similar range of responsive GC is seen in Figure A5, where the maximum log-expression seen for a bead-type (across the 250ng experiment) is plotted against GC content.

As the number of beads increases, so the precision of our observations increases, and so it is easier to achieve highly significant p-values. As can be seen in Figure A5, this trend is evident, but bead-types with observations that have very low numbers of beads are also more likely to return significant p-values (presumably because of unreliable estimates arising from such array-sections). The transition between the two trends appears to occur when the minimum number of beads seen is approximately 5.

7 Results: Detection

For each bead-type a detection score is calculated. Essentially this is an empirical p-value for the null hypothesis that there is no expression of the bead-type, and at its simplest is the proportion of negative control bead-types (that is, bead-types that should show no signal) that show higher expression than the bead-type in question. The one subtlety is that the set of negative control bead-types is pruned to remove those bead-types that appear to be showing expression themselves.

Taking a threshold of $p < 0.01$, we plot (for an individual array-section in each case) the numbers of bead-types (amongst the 21,627 in the analysis group) detected by each combination of starting RNA quantities. Note that for both 100% UHRR and 100% Brain the two most populous cells are concordant amongst all four quantities of starting RNA (present in all four, or absent in all four). The pattern for the next two cells is again the same for 100% UHRR and 100% Brain, with detection in all but the 10ng experiment being the third most populous cell, and detection in only the 250ng experiment fourth.

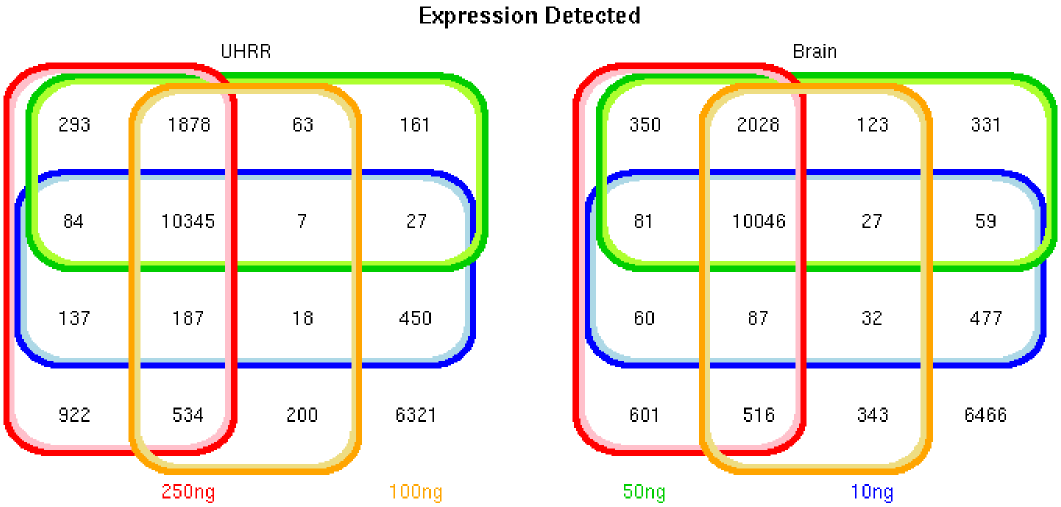

Figure A6: Venn diagrams illustrating the numbers of bead-types for which detection is expressed by each combination of the four experiments.

8 Results: Negative controls

There are 759 negative control bead-types on the array, and plotting the p-values for differential expression, we see that there is evidence for departure from the uniform distribution in favour of differential expression (Kolmogorov-Smirnov test: p = 0.0014).

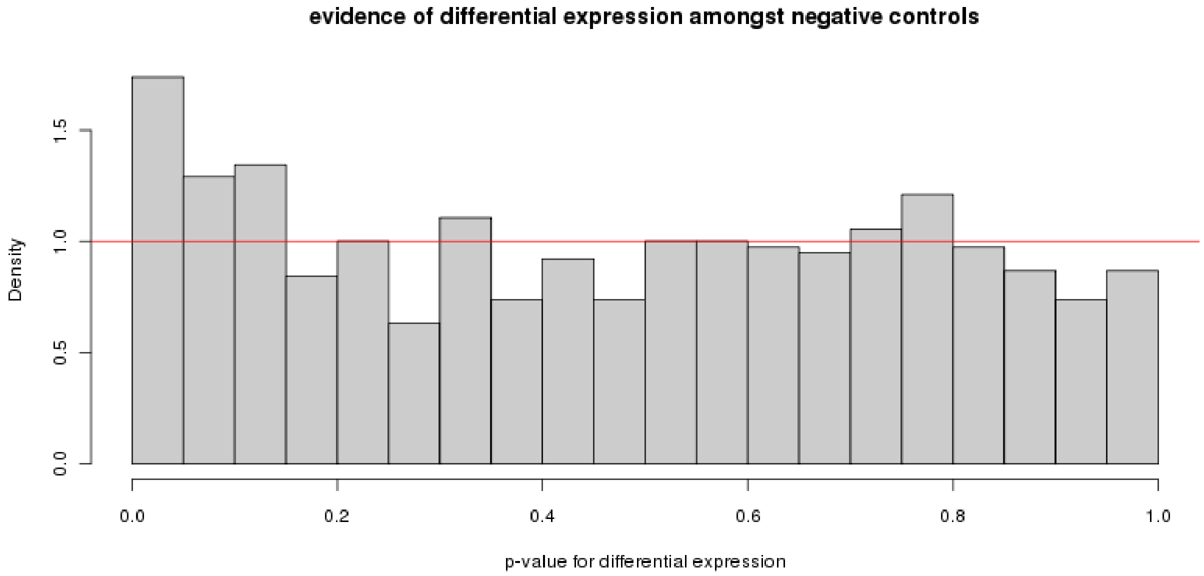

Figure A7: Histogram of p-values for evidence of differential expression from negative-control bead-types.

One particular negative-control bead-type (ILMN_1343923) shows strong evidence of being differentially expressed that is consistent across the different quantities of starting material. (250ng – black, 100ng – green, 50ng – orange, 10ng – red). Despite the evidence of expression in Figure A7, the sequence has no matches when alignments are sought against the human genome nor a number of transcript databases [see reference 16 of main manuscript]. There seem to be three possible explanations for this: a) that the reference genomes are incomplete, b) that there is content within the UHRR sample that comes from a different genome, or c) that there is hybridization to the decoding sequence (undisclosed) of the bead-type.

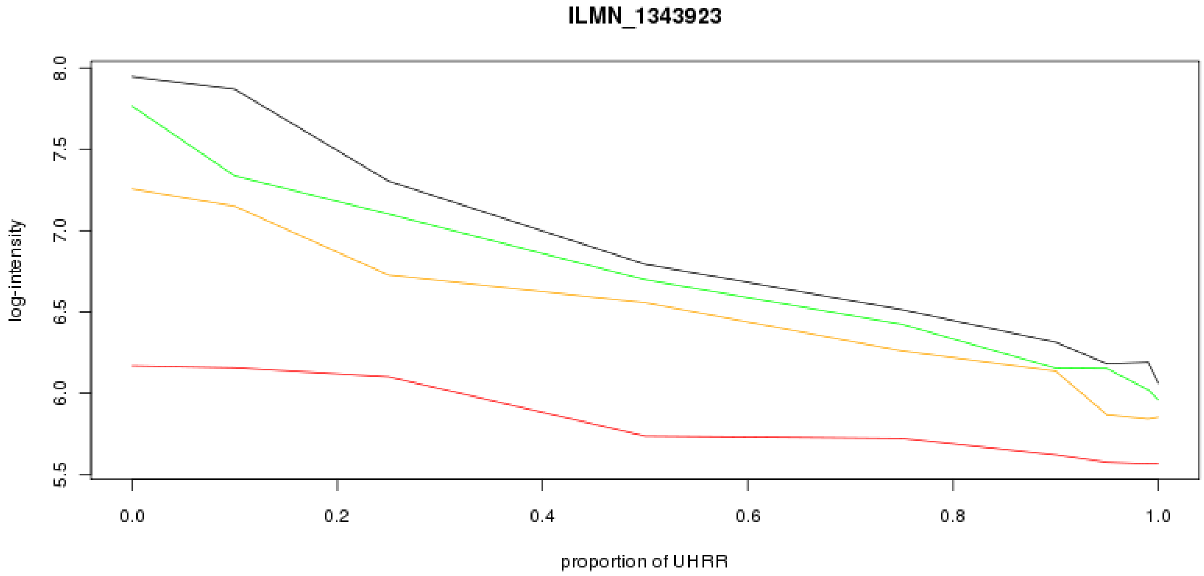

Figure A8: Expression profile for ILMN_1343923.

9 Results: Differential expression but no expression

For the 250ng experiment, there are 509 bead-types for which none of the 18 array-sections see expression detected at the significance level of 0.01, but for which differential expression is detected at a significance level of 0.001. That number decreases to 395, 286, and 109 as the starting quantity of RNA decreases to 100ng, 50ng, and 10ng respectively. While the concept of differential expression without expression is nonsensical, conceptually it is easy to see that a bead-type for which expression varies between two very low levels may never be picked up on an individual array-section, but that if the experiment is large enough, then the power will be present to detect differential expression.

An example of such a bead-type, ILMN_2100574, is given in Figure A9. As with Figure A8, the four experiments are indicated by colour (250ng – black, 100ng – green, 50ng – orange, 10ng – red). The 100ng experiment (green) comes close to being called as showing expression when the proportion of UHRR is 0.1, as one might expect from the figure, with a detection p-value of 0.0121.

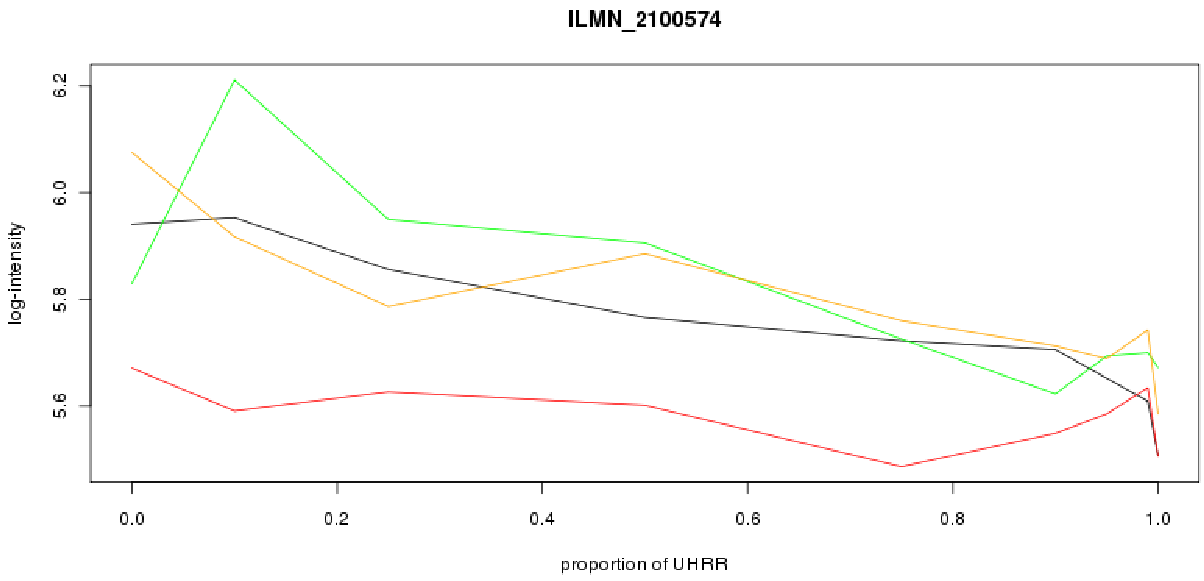

Figure A9: Expression profile for ILMN_2100574.

10 Results: Differential expression - detection of small changes

Here we illustrate how the use of more starting RNA enables the detection of more subtle changes in gene expression levels. We calculate the absolute difference in log- expression of Brain v UHRR in one of the starting quantities, and then plot this against categories of bead-types defined by the quantities of starting RNA at which they were detected as differentially expressed (Figure A9).

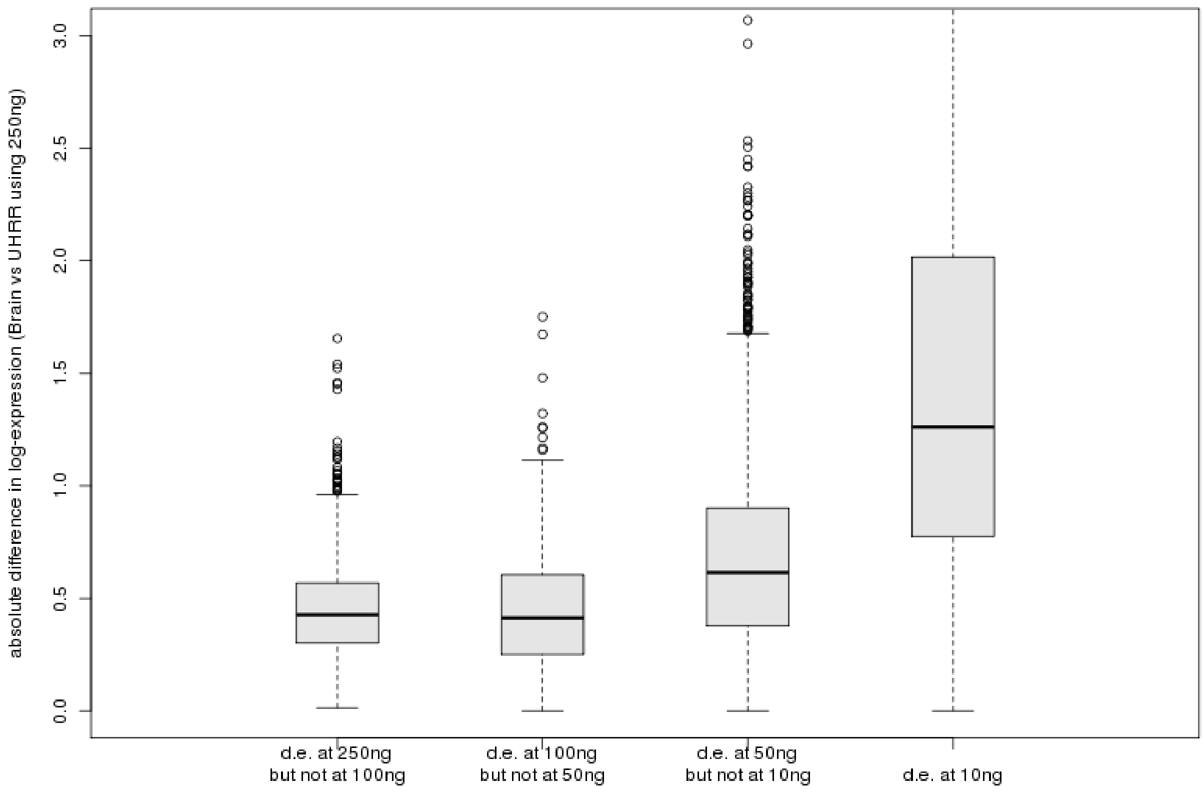

Figure A10: Illustrating the improvements in sensitivity to small changes in expression that are seen when the starting quantity of RNA is increased. (NB biased against the 250ng level)

In Figure A10 we see that the bead-types identified as differentially expressed by the 10ng experiment are generally large changes with a mean log-fold change of 1.56. The additional bead-types returned by increasing the starting quantity to 50ng (those detected as being differentially expressed at 50ng, but not at 10ng) are not a random subset, but tend to represent smaller log-fold changes. Again, when the starting quantity is increased to 100ng, the additional set of differentially expressed bead-types represents still smaller log-fold changes. This improvement is not seen in Figure A10 when the starting quantity of RNA is increased to 250ng, but this is because the representation is biased against the 250ng experiment as this is

the experiment used to calculate the y-axis values (we will be picking up anything that had a spuriously large log-fold change in the 250ng experiment). In Figure A11, we use the 100ng experiment to calculate the y-axis values, and with the bias reversed, we can conclude that the 250ng experiment does detect smaller log-fold changes than does the 100ng experiment.

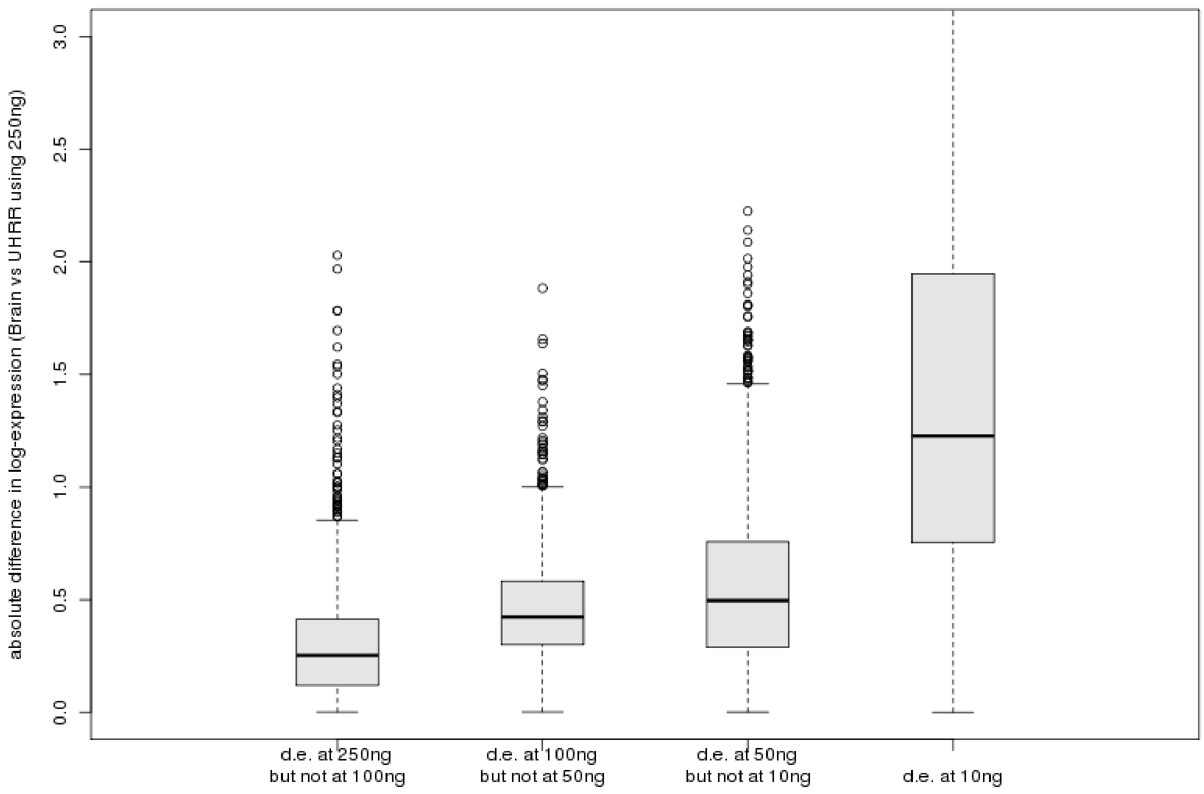

Figure A11: Illustrating the improvements in sensitivity to small changes in expression that are seen when the starting quantity of RNA is increased. (NB biased against the 100ng level)
